# Use of large language models for providing automated feedback in medical imaging education: a systematic review

**DOI:** 10.3389/fmed.2026.1803921

**Published:** 2026-06-10

**Authors:** Mustafa Mohammed Al-Mashhadani, Faika Ajaz, Shaista Salman Guraya, Farah Ennab

**Affiliations:** 1 College of Medicine (CoM), Mohammed Bin Rashid University of Medicine and Health Sciences (MBRU), Dubai Health, Dubai, United Arab Emirates; 2 Institute of Learning (IoL), Mohammed Bin Rashid University of Medicine and Health Sciences (MBRU), Dubai Health, Dubai, United Arab Emirates

**Keywords:** automated feedback, generative AI, large language model, medical education, radiology, systematic review

## Abstract

**Introduction:**

Large language models (LLMs) are an emerging form of generative artificial intelligence (AI) with promising applications in medical education, and their ability to provide automated feedback may enhance medical imaging education for trainees. This review aims to systematically examine and synthesize the published literature on the use of LLMs in providing automated feedback in medical imaging education.

**Methods:**

We conducted this systematic review in accordance with the PRISMA 2020 guidelines. A comprehensive search of the PubMed, Scopus, and Embase databases was conducted, covering studies published through January 2026. Our search strategy included keywords related to “feedback, generative artificial intelligence, large language models, radiology, and medical imaging.” Studies were eligible if they examined the use of LLMs to generate automated feedback for medical trainees within medical imaging education. Extracted data were synthesized using descriptive synthesis, with quality appraisal assessed using ROBINS-I and GRADE.

**Results:**

Of 1,003 identified records, 7 met the inclusion criteria. All studies examined the applications of automated LLM feedback in the medical education of radiology residents, with one study also including fellows. Reported educational outcomes included enhanced report quality, improved diagnostic accuracy, and increased efficiency in discrepancy detection. LLM feedback was generally well-received among trainees, with learners expressing satisfaction with the LLM feedback and preferring a hybrid human-AI feedback model. Additionally, fine-tuned models generally showed stronger performance than general-purpose LLMs and demonstrated variable agreement with expert-human consensus.

**Conclusion:**

LLMs show a potentially promising role as supportive tools for providing automated feedback in medical imaging education, alongside human feedback. This includes reported gains in accuracy, efficiency, and learner satisfaction. However, the current published evidence is preliminary and limited. Larger multicenter studies with standardized methods are necessary before widespread adoption can be justified. Our systematic review emphasizes that human expert oversight remains essential, as the current evidence supports preliminary technical feasibility, but not yet definitive educational effectiveness.

**Systematic review registration:**

https://www.crd.york.ac.uk/PROSPERO/view/CRD420251081394, Identifier CRD420251081394

## 1 Introduction

Medical imaging is a cornerstone of the modern medical environment, with reliable and accurate imaging linked to better patient outcomes (1). The availability of effective medical imaging has been shown to reduce mortality rates, improve diagnostic accuracy, and enable physicians to better understand patient presentations (1, 2). As medical imaging utilization increases in diagnosis, management, and follow-up across medical specialties, the ability to correctly interpret and communicate imaging findings has become a core clinical skill (3). This places a growing educational demand on medical trainees across the continuum, including medical students, residents, fellows, and those engaged in continuing medical education, to develop competence in imaging-related clinical reasoning (4). Medical imaging skills are also frequently perceived by trainees as difficult to learn, as they require integrating anatomical knowledge, spatial reasoning, pattern recognition, and clinical context, often without direct supervision (5). This perceived complexity emphasizes the need for focused and effective medical imaging education throughout training. Inadequate imaging education during the early phases of training may lead to reduced diagnostic confidence, inappropriate imaging requests, and misinterpretation of radiologic findings in clinical practice (6). In practice, the teaching of medical imaging varies across institutions and is often constrained by structural factors. Educational exposure may differ significantly depending on specialty, clinical rotation, and access to radiology teaching staff (7, 8). Imaging education is frequently delivered through lectures or opportunistic case discussions, with insufficient opportunity for structured assessment (9). Feedback, when provided, is often retrospective or inconsistent and may focus primarily on final answers rather than the underlying interpretive process, particularly in busy clinical settings (10, 11). These limitations may reduce learners’ opportunities to identify recurring errors or misconceptions, hindering skill acquisition and diagnostic accuracy (12).

Additionally, artificial intelligence (AI) services in healthcare have demonstrated notable improvements across multiple areas of medicine, including diagnostic accuracy, efficiency, and personalization of care (2, 13). In medical imaging, AI applications have primarily focused on image analysis and detection tasks, whereas more recent work has explored the role of Generative Artificial Intelligence (GenAI) in imaging workflows (14). The performance of GenAI in medical imaging reports is still being explored. GenAI can serve as an integrative component of the radiologist’s workflow, enabling prioritization of urgent cases, report standardization, and mitigation of fatigue-related errors (14, 15). These advancements have sparked curiosity about whether comparable technologies can be extended beyond clinical workflows to support educational processes. GenAI technologies are particularly useful in education because they can generate text-based responses, adapt output to user input, and provide explanations in natural language (16, 17). As a type of generative AI, Large Language Models (LLMs) can engage with open-ended prompts and free-text responses to draft sample reports, summarize imaging findings, and generate explanations tailored to the learner’s level (18). This ability to tailor feedback suggests that LLMs may support more personalized education by adapting explanation and feedback to individual learner needs, level of training, and patterns of error (19). Such applications suggest that LLMs may be well-suited to providing feedback on written imaging reports, differential diagnoses, and interpretive justifications when faculty time is limited. Beyond radiology, trainees across specialties also need support in image interpretation and appropriate imaging use, reinforcing the need for accessible and effective imaging education in medical school curricula (20).

Despite this necessity, current feedback practices in medical imaging education are frequently resource-intensive and reliant on expert availability (21). Faculty members may prioritize clinical responsibilities over teaching, especially in high-volume imaging departments, resulting in inconsistent or delayed feedback (22). These realities have prompted an interest in tools that could supplement rather than replace expert-led training. In this context, the growing popularity of LLMs in medical education, such as ChatGPT, has highlighted their potential to provide automated and actionable feedback in medical imaging education (23). LLMs can process narrative text and generate structured replies, making them useful for assessing written imaging reports, interpretive explanations, and clinical reasoning (18). Several recent studies have examined the use of LLMs to provide feedback on radiology reports, imaging interpretations, and structured reasoning tasks, suggesting that these models may offer learners timely and consistent feedback (24, 25). Some studies further considered learner perceptions of LLM-generated feedback, which was generally viewed as acceptable and useful by trainees (26, 27). Other potential benefits include immediate accessibility and the capacity to support repeated practice without increasing instructor burden (24). However, reported limitations include the risk of inaccurate or fabricated responses (i.e., hallucinations), limited awareness of clinical context, variability in alignment with expert opinion, and privacy concerns (15, 24). LLM performance is influenced by multiple factors, including the type of model used, fine-tuning, prompting strategies, and task design. In practice, because many evaluated systems are closed models, differences in performance across studies may reflect how the model was used and what educational task it was assigned, not only the data on which it was trained (28). Hence, appropriate safeguards and regulatory considerations are important in the context of AI in healthcare, with special consideration for integration with medical processes which would require such factors like risk mitigation, high-quality data, clear information, and human oversight as reported by the World Health Organization’s 2023 guidance (29).

An extensive search of the literature and review registration databases reveals that no previous systematic review has summarized the use of LLMs in providing automated feedback for medical trainees in medical imaging education. Existing reviews have primarily focused on diagnostic performance or general AI applications in radiology, without considering feedback as a separate educational outcome (30, 31). As a result, the current body of evidence is fragmented, making it difficult to draw conclusions about efficacy, limitations, and best practices. Therefore, the authors conducted this systematic review to evaluate the use of LLMs in providing automated feedback in medical imaging education, identify strengths and limitations of current models, and offer recommendations to inform future research and implementation.

## 2 Methodology

We used the Preferred Reporting Items for Systematic Reviews and Meta-Analyses (PRISMA) guidelines to guide the methodology of this review through all stages. The PRISMA guidelines serve as a checklist for evidence-based and standardized systematic reviews and meta-analyses.

### 2.1 Research objectives

The objectives of this review were designed with the Population, Intervention, Comparison/Context, Outcome, and Study design (PICOS) framework as shown in Box 1 (32). This systematic review aims to examine current practices reported in the published literature on the use of LLMs to provide automated feedback in medical imaging education. Specifically, the quality of LLM-generated feedback, the educational contexts in which LLMs are used (e.g., students, residents, fellows), reported educational outcomes, and learners’ attitudes and experiences with these tools. Together, these objectives help summarize and highlight the emerging best practices for implementing LLMs within medical imaging education.

BOX 1 **The PICOS framework table used for determining study inclusion during the title-and-abstract and full-text screening**

**Population**

Study involved medical imaging trainees (e.g., medical students, residents, fellows, continuing medical education, etc.)? YES/NO

*If NO exclude*

**Intervention**

Study uses Large Language Models (LLMs) (e.g., ChatGPT, Bard, Deepseek, custom-made LLM, etc.) for feedback?* YES/NO

*If NO exclude*

Is automated feedback provided by an LLM?* YES/NO

*If NO exclude*

*Feedback may include, but is not limited to: Discrepancy detection, error highlighting, diagnostic accuracy and suggestions, language/style critique, or/and teaching point generation.

**Context**

Study conducted in medical imaging educational context? YES/NO

*If NO exclude*

**Outcome**

Any reported educational outcome (subjective or objective)? YES/NO

*If NO exclude*

**Study design**

Original/empirical research (quantitative, qualitative, or mixed methods)? YES/NO

*If NO exclude*

### 2.2 Literature search strategy

We searched three major electronic databases (PubMed, Scopus, and Embase) for English-language publications until January 9th, 2026, with no search date restrictions. Initially, we searched the databases on June 23rd, 2025; however, we reran the search using the same strategy on January 9^th^, 2026, as the initial search was dated by then. We did not search grey literature (such as preprints and conference abstracts) as this review aimed to synthesize evidence from published empirical studies with sufficient methodological and reporting detail. The search strategy was developed by three reviewers (MA, FE, FA) by using Medical Subject Headings (MeSH) terms to optimize relevance while configuring Boolean AND/OR operators for advanced search. The authors were mindful of differences among database search engines; therefore, refined search strategies tailored to each database were developed. Search strategies were adapted for each database to account for indexing differences to balance sensitivity and specificity. Keywords and their variations used for this study include “Feedback, Generative Artificial Intelligence, Large Language Models, Radiology, and Medical Imaging.” The complete search strategy for each database is available in the registered protocol (CRD420251081394).

### 2.3 Study selection process

This review only included original and empirical research studies that (i) utilized LLM platforms and (ii) were related to medical imaging educational contexts and (iii) evaluated the educational outcomes of the LLM on trainee learning in medical education. Studies that did not focus on LLMs, automated feedback, education, or focused only on diagnostic performance were all excluded. A detailed inclusion and exclusion criterion was agreed and established in the study protocol and disseminated to all reviewers (MA, FA, FE) before commencing screening.

The selection process involved two stages. The initial stage included a title-and-abstract screening, and the second step involved a full-text review to determine inclusion or exclusion of the study from this review. Articles that enter the screening phases (title-and-abstract and full-text reviews) must receive two “Yes” votes from two blinded and independent reviewers (MA, FA), or one “Yes” and one “Maybe,” to move forward. Articles receiving two “No” votes were excluded, and any other combination was placed into a “Conflict” section, where a third blinded and independent reviewer (FE) made the final decision.

Covidence, a validated online platform for review management, was used to manage the references and selection processes for this review (33, 34). Covidence was used for title and abstract screening, full-text review, data extraction, blinding the reviewers, and reporting results per the PRISMA 2020 guidelines checklist, promoting comprehensive and transparent reporting of findings (33, 35). It was also used to automatically remove duplicates, and a PRISMA flow chart was generated by the platform (33, 35).

### 2.4 Data extraction and synthesis

Following the inclusion of studies after full-text screening, two reviewers (MA and FA) independently extracted data using the Covidence platform with a standard data extraction form designed to collect the necessary information (Box 2). Due to the heterogeneity of the included studies in terms of study design, LLM type, and outcomes, some studies did not report or evaluate certain data, so we transparently recorded irrelevant variables as “Not Reported” in our data extraction sheet instead of missing when applicable. We extracted all relevant information when possible and aimed to report our findings as transparently as possible in our Results section. Following independent extraction, the two reviewers (MA and FA) met to discuss similarities and differences, and any controversies or inconsistencies were resolved through discussion and a thorough comparison of the studies, including their inclusion criteria and reported publication data, until consensus was reached. The final conflict-free data was plotted on Excel and verified for accuracy by three reviewers (MA, FA, and FE). Extracted data from each included article contained study design, educational context, tool-specific information (e.g., LLM version, prompting strategies, type of feedback, etc.), and reported educational outcomes. We also extracted statistical results when reported in the included studies, reporting *p*-values when available.

A descriptive synthesis approach taking inspiration from published research was used to map and explore this review’s objectives (36). We employed a descriptive narrative approach for this review as we intended to capture and synthesize the heterogenous GenAI landscape in medical imaging education in a meaningful way. Though we never intended to perform a meta-analysis, we observed high heterogeneity in our small pool of included studies that encompassed different definitions and educational interventions under the broad LLM umbrella which further limited potential quantitative cross comparisons. The different reported methods and measured outcomes would have further limited meaningful direction of effect while statistical uncertainty would have limited applications of such results. Therefore, a descriptive narrative synthesis approach was undertaken to help manage reporting the high observed heterogeneity while limiting potentially misleading synthesis. The descriptive analysis started during the full-text review. As reviewers (MA and FA) started including articles for this review and becoming familiar with the literature, they noted some general concepts among the included articles. These concepts then developed a preliminary outcome section, outlining a general scope for the uses of LLMs in medical imaging education. As data extraction progressed, additional outcome groups were identified that had been missed initially. Following the data extraction, the heading concepts were reviewed, and all authors (MA, FA, SSG, and FE) read through the charted data and identified patterns. Using the outcomes, the authors refined and redefined the outcomes and generated overarching domains and subdomains within each. The organization, narrative, and presentation of the data were agreed upon in an iterative manner by all team members (SSG, FE, FA, MA) via group meetings that mapped how we communicated our findings. Finally, the synthesis used examples from the included studies and presented coherent, logical data aligned with the research objectives and the reviewed articles.

BOX 2 **Data extraction form used to capture outcomes and characteristics for each included study (*n = 7*)**

***Study characteristics***

Study Author

Year of Publication

Article Title

Journal Title

Country

Extra Comments

**Study design**

**Educational setting**

Educational Context (e.g., structured learning program, clinical rotations, clerkships, etc.)

Trainee Level

Sample Size

Additional Comments

**LLM usage**

LLM Name and Version

LLM Language

Prompting Strategy

Accuracy/Verification Checks

Type of Feedback Provided by the LLM

LLM and Tool-Specific Limitations

Additional Comments

**Educational outcomes**

Satisfaction with the LLM

Diagnostic Accuracy of the LLM

Time Saved by LLM usage

Perceived Accuracy of the LLM

Evaluation Metrics (Accuracy) of LLM Outputs

Feedback Preference (LLM vs. Traditional vs. Hybrid)

LLM Report Quality

LLM comparison to Expert Performance

Discrepancy Detection Performance by the LLM

Likelihood of Recommending LLMs for Automated Feedback

Reported Conclusions/Recommendations by the Authors

### 2.5 Quality assessment

This review critically appraised all included studies using the Risk of Bias in Non-randomized Studies of Interventions (ROBINS-I) (37). ROBINS-I was selected as the risk-of-bias evaluation tool because the majority of included literature was non-randomized and involved the intervention of an LLM. This framework enabled us to evaluate studies across domains for confounding variables, participant selection, intervention classification, deviations from intended interventions, missing data, outcome measurement, and selective reporting (37). This review further noted the funding sources and any conflicts of interest reported among the included literature. Although one study employed a randomized design, we retained the ROBINS-I appraisal tool for that study to preserve methodological coherence across this review, and all studies were evaluated against a common set of bias constructs that remain relevant in randomized studies. Additionally, we visualized findings from our ROBINS-I assessment via the web-based R programming environment *robvis* (38) to allow for a more practical presentation of study quality.

### 2.6 Quality assurance

This review further systematically assessed the quality of our evidence by utilizing the Grading of Recommendations Assessment, Development, and Evaluation (GRADE) framework (39). The GRADE assessment allowed us to determine the certainty of evidence for each key outcome by considering the number of studies reporting an outcome, study designs, risk of bias (informed by the ROBINS-I judgment), inconsistency, indirectness, imprecision, publication bias, and the overall certainty of the evidence for the outcome. Together, ROBINS-I and GRADE provided a transparent and structured evaluation framework for individual studies and the pooled narrative conclusions of this review.

### 2.7 Study registration

The protocol for this review was prepared and registered on a public systematic review protocol database (PROSPERO) before initiating the database search and screening. Prospective protocol registration allows our study to demonstrate transparency and reliability with our methodology throughout this review process. Although the registered protocol also specified the Cochrane Library, this database was not searched due to a lack of original primary studies, representing a deviation from the registered protocol. PROSPERO registration code: CRD420251081394.

## 3 Results

**Figure 1:**
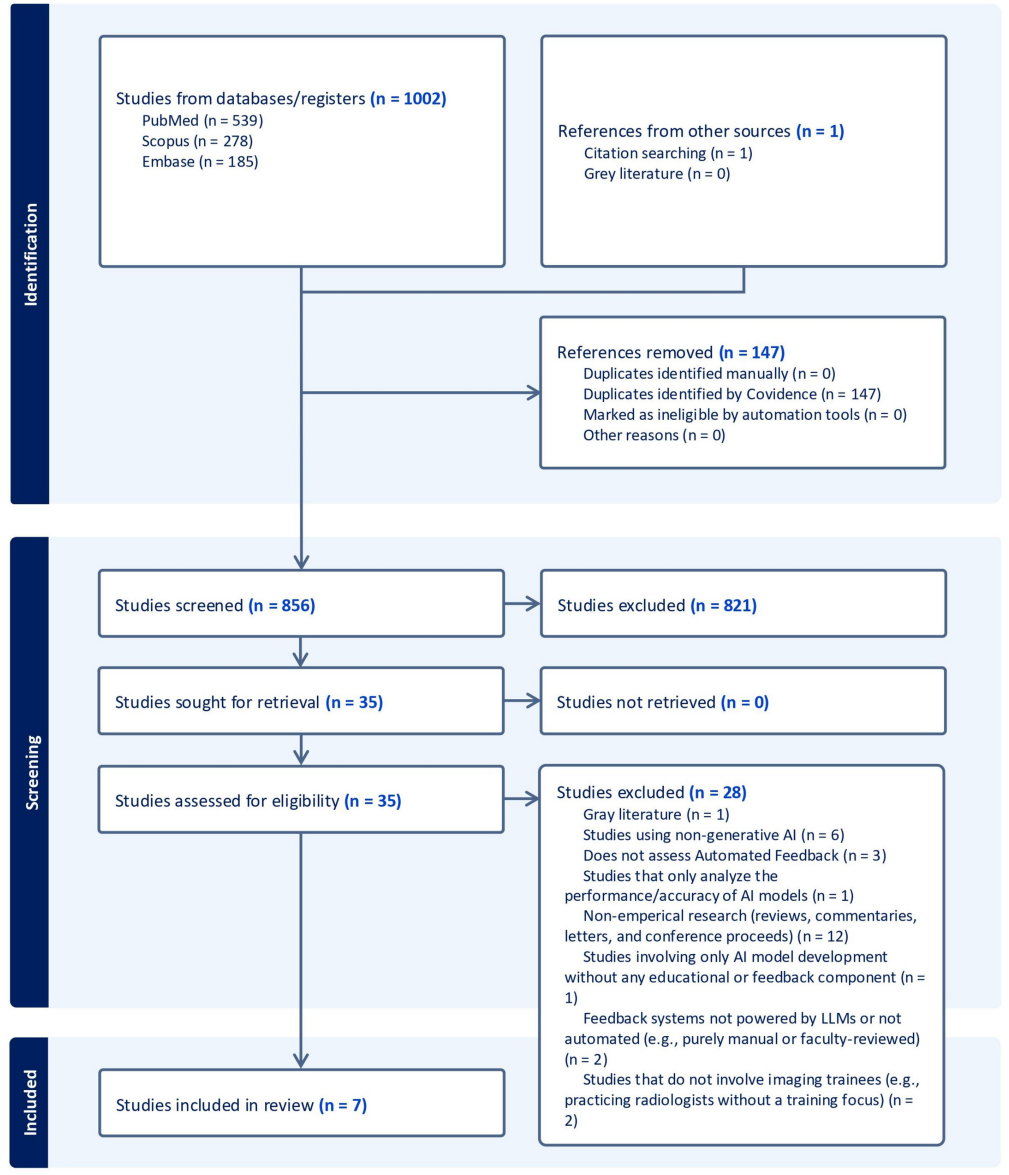
A PRISMA flow chart detailing the inclusion and exclusion process of studies from the initially identified 1,003 records (35).

Our search strategy yielded 1,002 studies across three databases (PubMed, Scopus, and Embase), with an additional paper imported into title-and-abstract screening following citation searching, bringing the total number of identified studies to 1,003. Covidence automatically removed 147 studies as they were duplicates. The authors conducted title and abstract screening on 856 studies, of which 821 were excluded at this stage. All 35 studies that entered the full-text review were successfully retrieved for the full-text, and 7 studies were deemed to match the study inclusion criteria. These 7 included articles (23, 26, 27, 40–43) were utilized in this systematic review. The study selection process is illustrated in Figure 1.

### 3.1 Studies characteristics

This systematic review captured LLM usage for automated radiology feedback encompassing 93 radiology residents and fellows, 28 radiology faculty, and at least 35,097 radiology reports. Sample sizes varied substantially across studies, ranging from small pilot cohorts of three radiology residents to large-scale analyses involving over 27,000 radiology reports. Key characteristics of the included studies are summarized in Table 1. All included studies discussed LLM automated feedback, with six studies being in the context of reports generated by radiology residents (23, 26, 40–43) and an additional study extending this focus to radiology fellows (27). The included studies evaluated multiple forms of automated feedback delivered by LLMs.

**Table 1:** Study characteristics of the included studies (*n* = 7) (23, 26, 27, 40–43), including author details, country, trainee level, imaging report modality, educational setting, LLM characteristics (model and feedback type), study design, and sample size.

Study (Year)	Country	Trainee level	Imaging modality of radiology reports	LLM & Version	Educational context	Design	Sample size
Alibrahim et al. 2025 (26)	Canada, Guyana	Residents	Computed Tomography (CT), X-ray, Ultrasound (US)	ChatGPT	Structured training program (residency)	Prospective cohort (within resident pre/post assessment).	3 radiology residents
Atsukawa et al. 2025 (40)	Japan	Residents	CT, Magnetic Resonance Imaging (MRI)	GPT-4 Omni (gpt-4o-2024–08-06)	Structured training program (retrospective report analysis)	Retrospective cross-sectional; LLM outputs compared with 2 board-certified radiologists.	9 radiology residents; 7,376 reports
Bala et al. 2024 (41)	USA	Residents	CT, MRI	GPT-4 (v4-0314)	Structured training program	Retrospective cross-sectional; LLM discrepancy flags validated against expert annotations.	14 radiology residents; 500 reports
Bartley et al. 2025 (42)	USA	Residents	CT, X-ray	RadBERT & comparators (Mistral, Llama2)	Structured training program	Retrospective cross-sectional; model outputs benchmarked to radiologist consensus review.	27,021 reports (10,335 head CT; 16,686 MSK radiographs)
Lopez-Rippe et al. 2025 (27)	USA	Residents & Fellows	CT, US, MRI	RADHawk	Simulation reporting	Prospective randomized controlled; tool-assisted reporting vs. usual resources; faculty grading.	57 radiology residents and fellows (*n* = 28 intervention, *n* = 29 control)
Lyo et al. 2025 (23)	USA	Residents	X-ray, unspecified neuroradiology reports	GPT-4 Turbo API (1104-preview)	Simulation reporting	Experimental evaluation; LLM outputs compared with radiologist reference labels/ratings.	200 reports
Zhou et al. 2025 (43)	Canada	Residents	Not reported	ChatGPT (GPT-3.5)	Structured training program	Comparative experiment; GPT-generated vs. faculty feedback; raters tested source discrimination.	28 faculty, 10 radiology residents

Discrepancy detection, including identification of missed findings through comparison of trainee and attending reports, was a prominent focus across several studies (23, 40–42). Educational or academic feedback, such as the provision of directions to relevant learning resources, was also commonly reported (23, 27, 42), whereas language and stylistic critiques of radiology reports were examined in a subset of studies (23, 26, 43). LLM-generated suggestions for additional differential diagnoses were also assessed (26, 40, 41), as was narrative feedback aimed at improving reporting time, such as strategies to reduce reporting time (27, 40). With respect to geographical distribution, the majority of included studies were conducted in the United States of America (23, 27, 41, 42), with additional studies conducted in Canada (43), Japan (40), and Guyana in collaboration with a Canadian institute (26).

Throughout this review, the term “LLM” is used only when referring to generative language or multimodal language models. To improve clarity, evaluated AI tools were categorized according to model type and intended use. General-purpose proprietary generative models included ChatGPT, GPT-3.5, GPT-4, GPT-4 Turbo, and GPT-4o. General-purpose open-source generative models included Llama 2 and Mistral. Domain-adapted or radiology-specific language models included models such as RadBERT, while radiology-specific task-oriented AI systems or pipelines included systems such as RADHawk.

### 3.2 Quality appraisal and assurance

**Figure 2:**
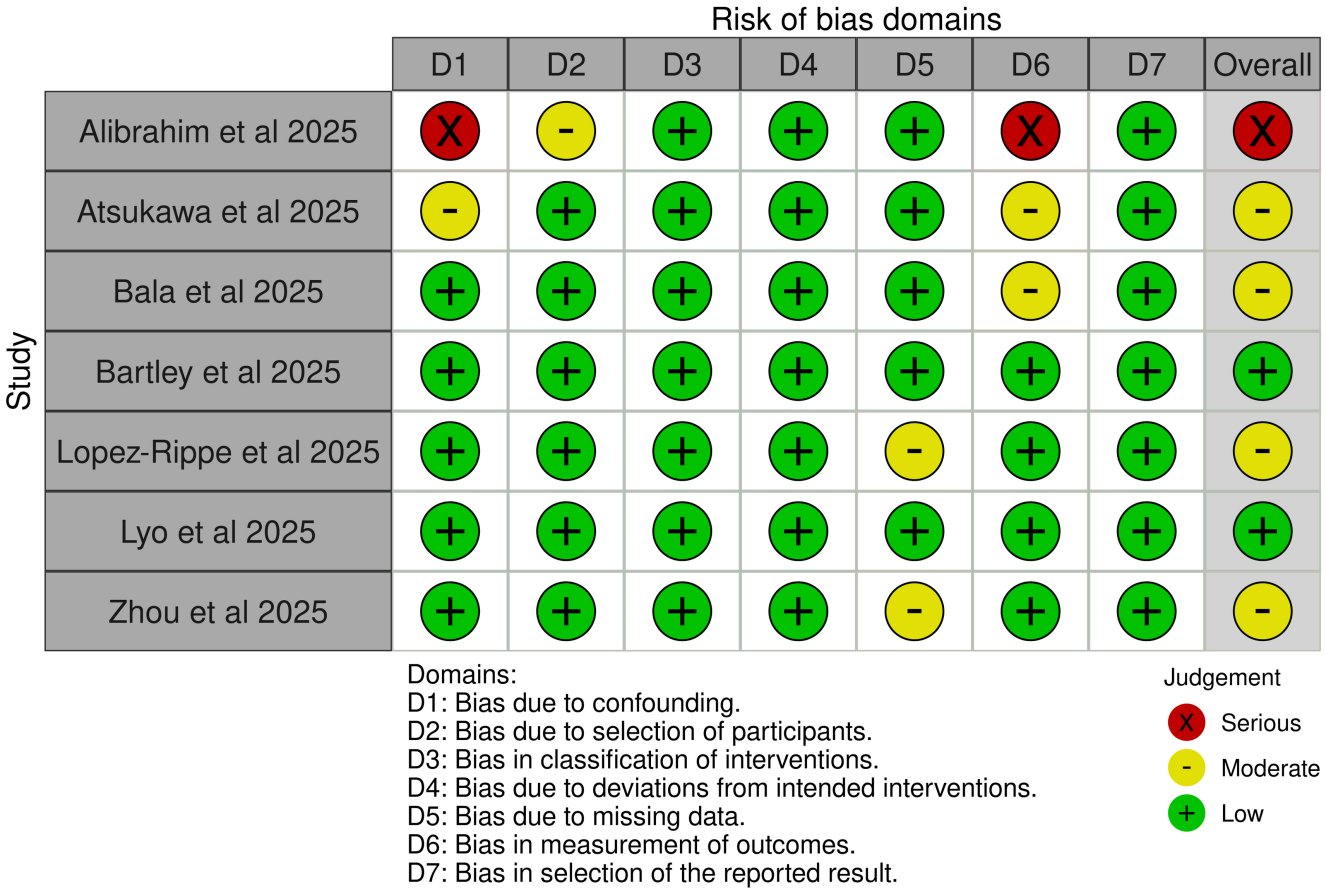
Study-specific assessment for risk of bias via ROBINS-I for all included studies (*n* = 7) (23, 26, 27, 40–43).

Quality appraisal was undertaken using ROBINS-I and GRADE. Figure 2 summarizes findings from our ROBINS-I assessment, while detailed breakdown of our GRADE assessment may be found within the Supplementary material. Based on the final ROBINS-I judgments, two studies were at low overall risk of bias (23, 42), four at moderate risk (27, 40, 41, 43), and one at serious risk of bias (26). The domains handled best across included studies were classification of interventions (D3), deviations from intended interventions (D4), and selection of the reported result (D7). Confounding was better controlled in studies with randomization, such as Lopez-Rippe et al. (27), or clear internal comparison and validation designs, such as Bala et al. (41), Lyo et al. (23), and Zhou et al. (43). Similarly, selection bias was reduced when reports or participants were randomly drawn from a defined source population (40–42). In contrast, the main weaknesses came from studies with no true comparator, very small convenience samples, or limited control of case-mix and outcome assessment. Alibrahim et al. (26), for example, was judged serious overall because the absence of a control group while assessing only three residents from a single institution made it difficult to separate the intervention effect from Hawthorne effects, while outcome measurement relied on self-reported Likert and quiz data susceptible to response bias. Atsukawa et al. (40) was downgraded because improvement over time may have reflected case complexity differences and outcome verification may not have been fully blinded. Bala et al. (41), Lopez-Rippe et al. (27), and Zhou et al. (43) were rated moderate, each limited by one domain-level concern such as unblinded review, participant exclusions, or insufficient detail about missing data. These ROBINS-I findings align with the GRADE assessment of low to very low certainty. Practically, for this review and for those designing future GenAI feedback interventions in education, the strongest lessons are that studies perform better when they include randomized or clearly defined comparison structures, objective expert-anchored outcomes, transparent intervention protocols, and complete reporting, whereas designs are weakened when they rely on single-site convenience samples, uncontrolled pre/post comparisons, subjective self-report outcomes, non-blinded assessors, or poorly described exclusions and missing data.

### 3.3 Descriptive synthesis

Across the seven included studies, reported educational outcomes were clustered into four major domains. Model- or task-level outcomes included discrepancy detection, agreement with expert labels, report-quality scores, and diagnostic accuracy against reference standards. Learner-level outcomes included changes in trainee report accuracy or performance over time, while system-level outcomes included workflow efficiency, time saving, perceived workload, satisfaction, and acceptability. Although outcomes were grouped into overarching domains to improve interpretability, variation in study design, sample size, model type, and outcome measures limit direct comparability across studies. Therefore, the findings below are interpreted as preliminary evidence of technical feasibility, acceptability, and selected performance improvements, rather than definitive evidence that LLM-generated feedback improves trainee learning.

The first and most extensively examined domain focused on report quality and accuracy, with six studies evaluating comparison to expert performance (23, 27, 40–43), four studies examining diagnostic accuracy and discrepancy detection (23, 27, 41, 42), and two studies reporting on overall report quality (27, 40). The second domain addressed trainee perceptions and feedback preferences, including satisfaction with the LLM model (26, 27, 41), perceived accuracy of LLM-generated feedback by trainees (23, 26, 41), preference for LLM-based versus traditional feedback (26, 41), and likelihood of recommendation or continued use (27, 41). The third domain captured outcomes related to workflow integration and time use, with two studies assessing time saved or workflow-related efficiency gains (27, 40). The fourth domain encompassed study-reported challenges, including limitations related to model performance implementation constraints, and the need for human oversight (23, 26, 40–43). A detailed comparison of these educational domains and their associated findings across studies is presented in Table 2.

**Table 2:** Overview of educational outcomes associated with LLM- generated automated feedback in medical imaging education.

Educational domain	Educational subdomain	Observed effect	Representative studies	Study-specific quantitative findings
Report quality and diagnostic performance	Report quality	Improved completeness and overall quality of trainee reports, reflected by fewer required revisions and higher accuracy/quality scores.	Atsukawa et al. (40)	Trainees received GPT-4o-generated feedback on radiology reports using a 6-criterion report-quality rubric (C1-C6); revision rates fell over time, especially fewer missed positive/negative findings and fewer deletions (C1-C3).
			Lopez-Rippe et al. (27)	RADHawk improved trainee report accuracy and quality, outperforming the control group by approximately 14% (*p* < 0.001)
	Diagnostic accuracy and discrepancy detection	LLMs identified missed diagnoses/discrepancies with moderate-to-high performance; sensitivity and error profiles varied by discrepancy severity and task/model.	Bala et al. (41)	GPT-4 reviewed 10 trainee radiology reports; flagged 24 potentially missed diagnoses; residents judged most flags as valid; overall sensitivity/accuracy 79.2%.
			Bartley et al. (42)	RadBERT classified discrepancy presence and severity against expert labels; high overall accuracy (90.5%); sensitivity increased as discrepancy severity increased (*p* < 0.005).
			Lopez-Rippe et al. (27)	Post-assessment diagnostic accuracy improved versus control (*p* < 0.001).
			Lyo et al. (23)	GPT-4 Turbo evaluated discrepancy detection using synthetic cases and the ReXVal dataset; detected more discrepancies than experts (*p* < 0.001); moderate-to-strong correlation with expert ratings; low false positive (FP) and false negative (FN) rates, with some issues attributed to labeling and rater-instruction differences.
	Comparison to expert performance	Higher agreement with experts for objective, structured criteria; mixed performance and lower agreement for nuanced clinical interpretation and narrative feedback.	Atsukawa et al. (40)	GPT-4o feedback aligned better with experts on structured/objective rubric items (C1, C2, C6) than on interpretive or style-related items (C3-C5).
			Lyo et al. (23)	Moderate agreement on discrepancy type and severity (~64%) between GPT-4 Turbo and radiologists; severity agreement higher than type.
			Zhou et al. (43)	GPT-3.5 narrative feedback compared with human expert feedback; humans were better at identifying comment origin; AI-human agreement on feedback quality was low.
Workflow Integration and Time Use	Time saved	Reduced reporting time per case in LLM-supported workflow	Lopez-Rippe et al. (27)	Reporting time reduced by ~162 s per case versus control (*p* = 0.002).
Trainee perception and feedback preference	Satisfaction with the model	Generally favorable usability and acceptance, though satisfaction ranged from modest to high depending on implementation and setting.	Alibrahim et al. (26)	High satisfaction and usability on Likert-scale ratings (4.3 ± 0.6); interaction generally smooth (mean 4.1 ± 1.1).
			Bala et al. (41)	Mean satisfaction ~3.5/5 on Likert-scale ratings (*p* = 0.04).
			Lopez-Rippe et al. (27)	RADHawk; high perceived ease-of-use (85.7%) and usefulness (78.6%); reduced workload domains versus control (e.g., mental demand, effort, frustration; all *p* = 0.03).
	Perceived accuracy of the LLMs by trainees	Trainees generally rated LLM outputs as accurate and relevant; teaching-point relevance tended to increase with clinically significant discrepancies.	Alibrahim et al. (26)	Information perceived as accurate (mean 4.4 ± 0.7) and sufficient to answer questions (4.2 ± 1.0).
			Bala et al. (41)	GPT-4 feedback was perceived as moderately accurate; mean score of 3.64/5.
			Lyo et al. (23)	GPT-4 Turbo teaching points; 84.5% rated relevant and relevance increased with discrepancy severity (Spearman *ρ* = 0.76, *p* < 0.001).
	Feedback preference (traditional vs. LLM)	Preference tended toward hybrid use (LLM plus traditional feedback) rather than LLM-only; preferences varied by comparator and task.	Alibrahim et al. (26)	Mixed preferences versus instructors/search engines; LLM preferred for some specific details but not consistently overall.
			Bala et al. (41)	Preference favored combined feedback; 71.43% preferred combined (LLM + traditional), 21.43% preferred traditional-only, 7.14% unsure.
	Likelihood of recommendation and future prospects	Neutral-to-positive willingness to recommend and continued interest in future access, indicating openness to adoption with appropriate safeguards	Bala et al. (41)	Likelihood of recommendation modestly positive (*p* = 0.03); 64.29% neutral, 21.43% recommend, 14.29% strongly recommend.
			Lopez-Rippe et al. (27)	Interest in continued use was high; 89.3% requested RADHawk access for their next rotation.

#### 3.3.1 Report quality and diagnostic performance

Most studies included evaluated educational outcomes across the following domains: report quality, diagnostic accuracy, discrepancy detection, and comparison to expert performance.

##### 3.3.1.1 Report quality

Atsukawa et al. (40) observed significant improvements in resident reporting skills over time, with the rate of LLM-initiated revisions dropping notably for missing positive findings (*p* < 0.001), unnecessary findings (*p* = 0.023), and missing negative findings (*p* = 0.004); however, because these findings were derived from single-arm longitudinal assessment, they should be interpreted cautiously, as improvement over time may also reflect natural trainee progression. Similarly, Lopez-Rippe et al. (27) reported a statistically significant increase in report accuracy among residents receiving LLM-generated feedback (*p* < 0.001) compared to controls.

##### 3.3.1.2 Diagnostic accuracy and discrepancy detection

Several studies reported higher diagnostic accuracy and discrepancy detection performance in settings where LLM-generated feedback or LLM-based discrepancy assessment was used; however, direct comparison across studies was limited by differences in ground truth definitions, evaluation metrics, and labeling approaches. Lopez-Rippe et al. (27) demonstrated a 14% increase in diagnostic accuracy in the intervention group (*p* < 0.001). Bala et al. (41) reported that the LLM used achieved an accuracy rate of 79.2% in identifying potentially missed diagnoses in trainee preliminary reports. Moreover, when compared to expert references, LLMs showed high efficiency in discrepancy detection. Bartley et al. (42) showed that the LLM achieved an overall accuracy of 90.5%, with model sensitivity increasing as discrepancy severity increased. According to Lyo et al. (23), the LLM used detected significantly more discrepancies in synthetic reports than radiologists (*p* < 0.001), with a moderately strong positive correlation between AI and human detection. Additionally, performance varied by model type. Studies using fine-tuned, task-specific models, including RadBERT (42) and RADHawk (27), reported higher accuracy and sensitivity metrics compared with studies evaluating general-purpose LLMs, such as ChatGPT, GPT-4o, GPT-4, GPT-4 Turbo and GPT-3.5 (23, 26, 40, 41, 43).

##### 3.3.1.3 Comparison to expert performance

Agreement between LLM-generated feedback and expert assessment was evaluated across multiple studies. Expert reference standards varied and included evaluations by board-certified radiologists, neuroradiology subspecialists, senior residents, and faculty consensus panels.

Atsukawa et al. (40) used six evaluation criteria to compare GPT-4o’s report revisions against those made by two board-certified radiologists, reporting substantial agreement for standardized tasks (e.g., addition of missing positive findings, *κ* = 0.72) and only fair agreement for more interpretive tasks (e.g., correction of interpretation of findings, κ = 0.29). Bala et al. (41) compared the LLM’s discrepancy detection to final reports from attending physicians, with accuracy confirmed by fourteen residents and false negatives validated by three senior residents. Bartley et al. (42) established an expert benchmark through blinded grading of report discrepancies on a 5-point scale by a board-certified radiologist, with subspecialist consensus review. Lopez-Rippe et al. (27) compared trainee performance using RADHawk to a control group, grading accuracy against faculty-generated reports, and observed statistically significant improvements for the intervention group. Lyo et al. (23) assessed GPT-4 against three neuroradiology subspecialists and reported moderate agreement between the LLM and radiologists, while noting that the model identified more discrepancies according to the study’s evaluation criteria. Although false positives and false negatives were observed, the majority were attributed to a single rater’s misinterpretation of instructions (23). As such, the apparent difference between LLM and human discrepancy detection in this study may partly reflect methodological factors rather than clear model superiority (23). In contrast, Zhou et al. (43) discovered that human raters outperformed GPT-3.5 in differentiating between AI and human-written comments. The inter-rater agreement between GPT-3.5 and humans was significantly lower (*κ* = −0.237) than the moderate agreement among people (κ = 0.502).

#### 3.3.2 Trainee perceptions and feedback preferences

Trainee perceptions of LLM-generated feedback were assessed across several studies, including satisfaction, perceived accuracy, feedback modality preference, and likelihood of future use. These outcomes were primarily assessed using post-intervention surveys and Likert-scale measures and therefore represent subjective perceptions, which may not necessarily correspond to objective improvements in diagnostic performance or learning outcomes.

##### 3.3.2.1 Satisfaction with the model

Across included studies, trainees generally reported positive experiences with LLM-generated feedback. Alibrahim et al. (26) reported high satisfaction and usability on Likert-scale ratings (4.3 ± 0.6), and interaction with the LLM was described as seamless (mean 4.1 ± 1.1). Bala et al. (41) similarly reported positive satisfaction outcomes, with a mean satisfaction score of approximately 3.50 out of a 5-point Likert scale (*p* = 0.04). Lopez-Rippe et al. (27) reported high perceived usability of the RADHawk system, with 85.7% of trainees rating it as easy to use and 78.6% rating it as useful. In addition, statistically significant reductions were observed in workload-related domains, including mental demand, effort, and frustration (all *p* = 0.03), in the intervention group compared with controls (27).

##### 3.3.2.2 Perceived accuracy of the LLM feedback

Perceived accuracy of LLM-generated feedback was rated highly by trainees across studies. Alibrahim et al. (26) reported that trainees found LLM-provided information as accurate (Likert scale mean 4.4 ± 0.7) and sufficient to answer questions (mean 4.2 ± 1.0). Bala et al. (41) reported that GPT-4 feedback was perceived as moderately accurate, with a mean score of 3.64 on a 5-point Likert scale (*p* = 0.01). Lyo et al. (23) also reported that 84.5% of LLM-generated teaching points were rated as relevant by expert raters (attending radiologists), with relevance increasing alongside discrepancy severity (Spearman *ρ* = 0.76, *p* < 0.001).

##### 3.3.2.3 Feedback preference (traditional vs. LLM) and likelihood of recommendation

Preferences regarding feedback modality varied across studies but generally favored hybrid approaches. Alibrahim et al. (26) reported that trainees had a partial inclination toward using LLMs for retrieving specific report details, with a mean preference score of 3.6 ± 0.6. However, preferences for LLMs over traditional faculty feedback or search engines decreased from pre- to post-intervention, though this finding was not statistically significant (*p* = 0.423) (26). Bala et al. (41) reported that 71.43% (10/14) of trainees preferred combined feedback, integrating LLM-generated and traditional faculty feedback. In contrast, 21.43% (3/14) preferred traditional feedback alone, and 7.14% (1/14) reported uncertainty. In the same study, likelihood of recommending LLM-generated feedback was neutral to positive, with a mean recommendation score of 3.5 ± 0.73 (*p* = 0.03) (41). Within this group, 64.29% of trainees were neutral, 21.43% recommended, and 14.29% strongly recommended LLM-generated feedback (41). Lopez-Rippe et al. (27) reported high interest in continued use, with 89.3% of participants requesting access to RADHawk for their next rotation.

#### 3.3.3 Workflow integration and time use

Among the included studies, only Lopez-Rippe et al. (27) objectively evaluated workflow efficiency and time use following LLM integration. The authors reported a statistically significant reduction in case report time by 162 s per case in the intervention group compared to the controls (*p* = 0.002). In the same study, cognitive workload was assessed using the validated NASA Task Load Index (NASA-TLX) survey, which demonstrated lower perceived cognitive load and stress on trainees in the intervention group (27). In contrast, Atsukawa et al. (40) did not report an objective measure of time use or workflow efficiency. Workflow integration outcomes were instead described subjectively, with the authors suggesting that LLM-generated feedback may reduce workload for both trainees and supervising radiologists (40). No validated surveys or quantitative measures specifically assessing time use, cognitive load, or workflow efficiency were reported (40). Since workflow-related outcomes were evaluated objectively in only one included study and were not assessed consistently across the literature, conclusions regarding efficiency gains should be interpreted as preliminary.

#### 3.3.4 Study-reported challenges

The included studies noted recurring challenges related to LLM performance and implementation. These limitations were reported to affect reliability, implementation, and clinical nuance of LLM-generated automated feedback.

Hallucinations and output reliability were frequently noted. Alibrahim et al. (26) reported an inherent risk of hallucinations particularly when prompts were generic or exceeded the scope of the training dataset. Zhou et al. (43) observed repetitive outputs that lacked contextual or educational diversity when models were trained on narrow datasets, and further noted poor performance by the LLM in differentiating between AI and human remarks. In addition, technical and implementation issues were also described. Alibrahim et al. (26) reported delayed or absent responses and integration challenges when LLMs were used alongside existing technologies, such as issues with synchronizing with virtual reality sets or pointers. Bartley et al. (42) demonstrated that general-purpose LLMs showed lower accuracy compared with task-specific, radiology fine-tuned models. Lyo et al. (23) reported persistent false positives and false negatives due to an over-reliance on text-only inputs, a drawback in radiological settings where visual context may be needed.

A common limitation across studies was reduced clinical nuance and contextual reasoning. Atsukawa et al. (40) reported lower agreement for interpretive and expression-based rubric items compared with more structured or objective items. Bala et al. (41) similarly noted weaker performance for subtle or context-dependent findings, while Alibrahim et al. (26) reported that current LLM implementations may not adequately assess higher-order skills such as critical thinking. Zhou et al. (43) observed that AI-generated narrative feedback was perceived as less nuanced and less personalized than faculty feedback, and was often overly formal in tone, lacking empathetic elements present in human feedback. Error rates and discrepancy assessment further limited reliability. Bala et al. (41) reported that their model missed 20.8% of diagnoses, while multiple studies described tendencies toward over-calling discrepancies or overestimating discrepancy severity relative to expert assessment (23, 41, 42). Finally, governance and oversight concerns were commonly cited. Several studies raised concerns regarding data privacy and ownership, potentially limiting institutional adoption of commercially available LLMs (23, 42). Consistent with these findings, multiple authors emphasized that LLM-generated feedback should supplement rather than replace expert supervision, with Bala et al. (41) reporting trainee preference for hybrid feedback models and Zhou et al. (43) demonstrating inferior performance of GPT-based models compared with human experts for detailed narrative feedback.

## 4 Discussion

Our systematic review captured the current published applications of LLMs in providing automated feedback for medical imaging education via a narrow yield of 7 original studies. The studies reviewed suggest that LLMs have preliminary potential as adjunctive tools for automated feedback in medical imaging education; however, the current evidence more strongly supports technical feasibility, usability, and selected short-term performance outcomes than definitive educational effectiveness. Across the included studies, LLMs demonstrated promising model- or task-level performance in areas such as discrepancy detection, expert agreement, and report-quality assessment, while trainees generally reported favorable perceptions of usability and acceptability. However, these outcomes should be distinguished from learner-level educational effectiveness, which would require stronger evidence of sustained improvement in trainee knowledge, diagnostic reasoning, reporting performance, retention, or transfer to independent clinical practice. Therefore, the findings should be interpreted as early and hypothesis-generating rather than conclusive. Despite this, the current literature points to certain advantages that LLMs may offer in the medical imaging educational context. To start, the usage of LLMs for automated feedback may be affordable and effective, enabling usage in resource-limited settings (26). LLM generated automated feedback was reportedly accurate and well-received by trainees, who preferred a hybrid strategy that combined AI and traditional faculty-provided feedback (41). Some objective benefits were further described, with reduced reporting time, enhanced reporting accuracy, and decreased overall cognitive burden for radiology trainees (27). These models can also objectively assess revisions in radiology resident reports and may potentially be able to track reporting skill development over time, particularly for standardized report elements (like addition or deletion of findings), but are less reliable for more subjective or context-dependent tasks (40). LLMs may also accurately detect both minor and major report discrepancies, with fine-tuned models performing better than general-purpose ones in selected tasks (42). In some studies, LLMs identified more report discrepancies according to study-specific evaluation criteria; however, greater discrepancy detection should not be interpreted as straightforward superiority over radiologists. In Lyo et al. (23), for example most false-positive and false-negative discrepancies were attributed to a single human rater’s misunderstanding of task instructions, indicating that the observed difference may reflect methodological factors rather than true model superiority. More broadly, variation in reference standards, evaluation metrics, and labeling approaches across studies limited direct comparability of reported diagnostic accuracy and discrepancy detection outcomes. In this subsequent section, we outline considerations from this review to guide and support the design of LLM interventions in imaging education.

### 4.1 Implementing LLMs for automated feedback

The included literature has exclusively studied a variety of LLMs and their applications for automated feedback among radiology residents, with one study also extending to fellows. Taken together, these findings suggest that the development of an LLM-based automated feedback intervention should be guided by (i) the intended educational task (e.g., discrepancy detection vs. narrative education), (ii) the imaging modality and subspeciality context, and (iii) the educational stage of trainees.

#### 4.1.1 Choice of LLM

Firstly, it is important to choose the most appropriate LLM for an intervention guided by the described goals. The included studies assessed both general-purpose models (e.g., ChatGPT), and fine-tuned radiology-specific models (e.g., RadBERT, RADHawk). Overall, fine-tuned models were reported to outperform general-purpose LLMs in providing feedback and showed closer agreement with expert-human consensus (27, 42). The patterns observed in this review support a practical approach to LLM utilization, with domain-specific or fine-tuned models performing best for structured tasks (e.g., discrepancy detection or case severity classification), and general-purpose LLMs for broader formative feedback (e.g., report or language phrasing, educational explanations, and targeted teaching points). Despite this, it is important to note that the quality and output of LLMs vary between models and prompt designs (44). Importantly, LLM reliability issues are also important to consider, with inaccurate or fabricated responses (“hallucination”) reported among the broader GenAI literature and included studies (26, 45, 46).

#### 4.1.2 Timing of the intervention

Because the current evidence is concentrated toward radiology residents, LLM utilization for medical imaging feedback appears most suitable during the training phase, when learners are actively producing preliminary medical imaging reports and can contextualize feedback against clinical practice and faculty supervision. A staged implementation may be optimal, beginning with simulations or other low-stakes training environments before expanding LLM feedback applications into routine clinical workflows, where patient safety considerations are imperative (23, 27, 47, 48). This would involve familiarizing learners with the model and helping them understand its strengths and pitfalls. This progressive introduction of the LLM intervention allows trained individuals to develop feedback literacy, learning how to interpret, verify, and act upon suggestions (49). The progressive introduction may also help reduce uptake of incorrect outputs by mitigating automation bias or over-reliance bias. which is particularly relevant considering the risks of hallucinations from GenAI (45, 50). As trainees progress with their learning, their needs also change. One of the key potential educational advantages of LLM-based systems is their capacity to provide more personalized or adaptive feedback by tailoring the level of explanation, type of guidance, and emphasis of feedback according to trainee stage, recurring errors, and task complexity (19). Junior trainees may benefit more from feedback that helps learners with structure, educational context guidance, and writing support, which may be offered by general-purpose LLMs. Inversely, more senior trainees may benefit from LLMs that report on efficiency and quality improvement (e.g., discrepancy detection), which may require more fine-tuned or specialized LLMs.

#### 4.1.3 LLM selection by modality and task

LLMs evaluated in this study encompassed various imaging modalities, including CT, MRI, radiographs, and ultrasound, and varied in the tasks they performed. In general, the evaluated LLMs did not stand out as particularly useful in one modality or another, but the effectiveness of their application was more relevant to the task and training context. For example, Bartley et al. described the potential advantage of modality and domain training via producing a radiology-oriented model trained in emergency medicine settings (RadBERT) to be advantageous in the objective of discrepancy classification for emergency reports, while performing poorly in other contexts (42). For broader multimodality educational support (for example, answering questions, explaining findings, and suggesting differentials), a more general-purpose LLM may perform more reliably and help achieve learning outcomes more efficiently. The findings suggest that educators should match the model to the educational objective, with discrepancy detection and triage more suitable for fine-tuned and context-trained models, whereas explanatory and broad teaching may be more suitable for general-purpose LLMs, provided that the LLMs are verified and align with departmental standards.

#### 4.1.4 Frequency and duration of the intervention

The intervention duration should be optimal to enable repeated practice and exposure, yet structured in such a manner that it avoids cognitive overload or overreliance. Evidence in the literature suggests that repeated exposure can improve learning outcomes. For example, Atsukawa et al. reported that the number of changes residents needed to make on their reports declined over time, indicating potentially improved performance in report writing (40, 51). This supports designs where LLM feedback is delivered per case, providing immediate feedback after each preliminary report while the case is still recent in the mind of the trainee, supplemented by periodic faculty review sessions that may consolidate lessons and recalibrate learner interpretations (52). This set-up may also allow trainees or faculty to summarize learning gaps via the LLM and revise pitfalls later on.

Duration should also align with the expected skill acquisition trajectories, as short or single-session exposure may demonstrate immediate usability and improvement, yet may lack in supporting long-term knowledge acquisition or behavioral changes such as retention or communication skills like those needed in multi-disciplinary team (MDT) meetings (53). Duration of the intervention should take into consideration rotation lengths and current trainee educational expectations while examining before and after intervention results (27). For example, Alibrahim et al. reported that through their 8-week LLM course, trainees were able to learn in a manner distributed along the week while demonstrating pre/post intervention score gains without requiring high-intensity or continuous learning modules (26).

Altogether, this suggests that the optimal duration of the intervention can often be rotation-length (around 4–8 weeks) with per-case LLM feedback and additional weekly/biweekly faculty sessions for summarization/recall. However, the optimal dose is not yet empirically established and likely varies by task and training level.

#### 4.1.5 Faculty oversight

Faculty oversight was integral to the higher-rigor studies included in this review, including direct grading, benchmarking of trainee outputs, and assessment of LLM feedback (42, 43). Studies reviewed highlight the ongoing preference of faculty guidance alongside LLM feedback, in addition to the need for supervision via the faculty. This emphasis is consistent with recent radiology education literature showing that students perceive faculty oversight as an important factor in providing a credible and trustworthy educational framework (54). Faculty oversight is necessary to (i) detect and correct hallucinations or unsafe suggestions (23, 40–42), (ii) ensure alignment with institutional reporting standards and safety (23, 40, 42), (iii) calibrate trainee learning and appropriateness of the feedback for the trainee’s level (40, 43), and (iv) reduce over-trust and automation bias (41). Given the heterogeneity and preliminary nature of the evidence, structured faculty governance and boundaries (LLMs as adjuncts rather than replacements) are central requirements for responsible implementation, rather than optional safeguards (23). However, how LLMs and faculty interact within these roles is not yet a fixed concept. The optimal balance between LLM educational autonomy and faculty oversight is highly context-dependent, shaped by, for example, the nature of the reporting task, the clinical setting, and the learner’s level of training. As such, defining the ideal human-in-the-loop framework remains a moving target that will likely evolve as models improve and institutional experiences accumulate. Multiple longitudinal studies are therefore needed to better understand how faculty oversight should develop alongside advancing LLM capability, and how to preserve its educational and safety functions without compromising the efficiency gains these tools may offer.

### 4.2 Implications and future direction

This review has identified several areas for improvement to advance the use of LLMs in radiology education. Firstly, although the PICOS framework intentionally adopted a broad educational scope that included medical students, residents, fellows, continuing medical education learners, and other educational contexts beyond radiology residency, the evidence captured in this review primarily focused on radiology residents, with only one study also including radiology fellows. As such, future studies may benefit from exploring learning outcomes among trainees beyond the context of radiology residency alone. Moreover, multiple authors emphasized the need for larger studies with diverse participant groups and control arms to validate findings and enhance generalizability (26, 27, 41, 42). To achieve greater scalability, external validation across multiple institutions was also recommended (27, 41, 42). Moreover, when handling complex cases and discrepancies, it was frequently advocated to fine-tune the models to enhance accuracy (23, 40–43). The authors also suggested using advanced prompting techniques and multimodal data, such as combining text and images, to maximize model outputs (23, 42). Lastly, for effective integration of AI and to ensure the safety and quality of education, it was considered essential to maintain human expert supervision throughout (41, 43). In real-world educational settings, this supervision could take the form of faculty review of selected cases, benchmarking of LLM feedback against expert consensus, and predefined thresholds for when AI-generated feedback should be accepted, revised, or escalated for direct human input.

This systematic review indicates that designing interventions using LLMs requires consideration of several pragmatic factors. Firstly, studies should employ objective outcome assessments and expert verification of feedback quality to ensure accurate and unbiased reporting. In addition, future research would benefit from greater standardization of outcome definitions and evaluation metrics as highlighted by this review, particularly for constructs such as report quality, diagnostic accuracy, discrepancy severity, feedback usefulness, and workflow efficiency, to improve comparability across studies and strengthen interpretation of findings. Future designs evaluating LLMs should also incorporate control groups via groups not receiving an LLM intervention, faculty-generated consensus material, or within learner pre/post-intervention assessments when randomization is not possible. The incorporation of standardized AI guidelines and established medical education frameworks would further strengthen methodological rigor (55). We found that the strongest educational designs incorporate (i) objective outcomes, (ii) blinded faculty, and (iii) pre-specified thresholds for acceptable performance and escalation. Future studies can also situate LLM-based interventions within the broader context of medical education research and enable insightful comparisons by linking research design and outcome assessment to commonly used frameworks such as Miller’s pyramid, feedback theory, and the simulation training literature (27, 56).

### 4.3 Strengths and limitations

This review rigorously searched three databases; however, the risk that studies were not captured by our search terminology, database coverage, and language restrictions remains. Grey literature, preprints, and conference abstracts were also not systematically searched, which may have excluded emerging work in the rapidly evolving LLM literature. Secondly, although we aimed to capture and synthesize the best practices for LLM usage, small sample sizes and heterogeneous methods made it difficult to attribute outcomes to interventions. In addition, the included studies measured outcomes at different evaluation levels, including model-level performance, learner perceptions, workflow efficiency, and limited learner-performance outcomes. This heterogeneity restricts the extent to which findings can be interpreted as evidence of educational effectiveness, particularly because model agreement, discrepancy-detection accuracy, satisfaction, and time saving do not necessarily demonstrate durable learning or improved independent diagnostic performance. Moreover, a GRADE assessment of the evidence extracted in this review indicated low to very low certainty, largely due to heterogeneity and limited sample sizes (see Supplementary material). Despite this, several studies reported directionally favorable findings related to feasibility, usability, and selected performance outcomes, although these should be interpreted cautiously given the low certainty of evidence. Our review may also introduce biases associated with only including 7 small-scale and single-institution studies, limiting the significance and transferability of the findings. The included studies were also short-term, so the long-term impacts of LLM use in education may not have been adequately validated. In addition, some included pre/post or longitudinal studies may have been confounded by natural trainee progression over time, which limits attribution of observed improvements solely to the LLM-based intervention. Finally, research on the use of LLMs across disciplines is progressing rapidly, therefore this review reflects a snapshot of an emerging and evolving body of work.

Although there remain some limitations, this review contributes to the emerging early applications of LLM-generated feedback in medical imaging education by consolidating and synthesizing the current evidence in a systematic manner. To our knowledge, this is the first review of its kind synthesizing study designs, model types, educational contexts, outcomes, and limitations to illustrate the current applications and boundaries of LLMs in medical imaging feedback. The findings provide our suggested implications for future research, including the need for objective outcome measures, expert verification, faculty oversight, staged deployment, and standardized reporting.

## 5 Conclusion

This systematic review suggests that LLMs may have a potential adjunctive role in medical imaging education by providing timely, automated, and standardized feedback to trainees. However, the current evidence base more strongly supports preliminary technical feasibility, acceptability, and selected short-term performance outcomes than definitive educational effectiveness. Across the included studies, LLMs showed promise in model- and task-level outcomes such as discrepancy detection, report-quality assessment, and agreement with expert benchmarks, and were generally perceived favorably by trainees, but the strength and educational meaning of these findings remain limited by study design, heterogeneity, and small sample sizes. Current applications remain situated within a still novel and rapidly evolving generative AI landscape. The included studies are characterized by small sample sizes, limited generalizability, and tool-specific constraints, which limit the ability of LLMs to replace expert feedback. Instead, existing applications are best understood as supplementary tools that support, rather than substitute, feedback processes in imaging education. To advance the role of LLMs in education, findings from our review suggest a need for larger, multi-institutional studies that incorporate model fine-tuning and integration strategies aligned with established educational and developmental frameworks. Finally, a hybrid approach that places LLM-generated feedback alongside human expert input may be optimal for creating trust, reliability, and in-depth educational experiences.

## Data Availability

The original contributions presented in the study are included in the article/Supplementary material, further inquiries can be directed to the corresponding author.
